# 

**Published:** 1860-04

**Authors:** 


### CONTENTS.

ORIGINAL ESSAYS AND COMMUNICATIONS.

The Physiology and Pathology of the Mouth.... 457

An Address at the Commencement of the Dental College of Ohio. By B. P. AYDELOTT, M. D., D. D.... 462

Irregularity—Treatment. By DR. GEO. LUPTON.... 476

PROCEEDINGS OF SOCIETIES.

Sixteenth Annual Meeting of the Mississippi Valley Association of Dental Surgeons..... 479

Sixteenth Annual Meeting of the Mississippi Valley Aassociation. Discussion.... 486

EDITORIAL.... 503

